# PSYCHOSOCIAL FACTORS AND EARLY MID-LIFE BLACK WOMEN’S SLEEP QUALITY: A CONSIDERATION OF INTERSECTIONAL MEASURES

**DOI:** 10.1093/geroni/igad104.0668

**Published:** 2023-12-21

**Authors:** Christy Erving

**Affiliations:** University of Texas at Austin, Austin, Texas, United States

## Abstract

Beyond an individual behavior, scholars are progressively conceptualizing sleep as embedded in social factors. Extant research establishes that sleep is influenced by experiences based on status characteristics such as gender or race along with psychosocial stressors such as discrimination. Moreover, sleep is important in the context of gender and racial disparities, as Black women experience relatively poorer sleep quality compared to their Black male and White counterparts. Given their relatively poor sleep quality, this presentation will center Black women by examining how psychosocial factors influence their subjective sleep quality in early mid-life, an important life course stage characterized by significant role obligations in personal and professional life domains. This presentation also takes an intersectional approach, examining the sleep implications of two recently developed measures that capture the unique intersectional oppression of Black women: Superwoman Schema and gendered racial microaggressions. To assess the association between these intersectional psychosocial factors and subjective sleep quality, data are drawn from Mechanisms Underlying Stress and Emotions (MUSE) in African-American Women’s Health Study, a cohort of 422 Black women (ages 30-46) in Atlanta, GA, USA. Preliminary findings suggest that both Superwoman Schema endorsement (e.g., feeling obligated to help others) and reports of gendered racial microaggressions (e.g., feeling marginalized and silenced in professional settings) are related to poor subjective sleep quality. Moreover, these associations remain even after adjusting for various factors including, but not limited to, depressive symptoms, anxiety symptoms, and social support. Study implications and future research directions will be discussed.

